# Development and validation of Competing Sentence Test in Kannada

**DOI:** 10.1590/2317-1782/20242023355en

**Published:** 2024-10-18

**Authors:** Bhavana Rhythm, Hari Prakash Palaniswamy, Arivudai Nambi Pitchai Muthu

**Affiliations:** 1 Department of Speech and Hearing, Manipal College of Health Professions, Manipal Academy of Higher Education, Manipal, India.; 2 Department of Audiology, Centre for Hearing Science, All India Institute of Speech and Hearing - Mysuru, India.

**Keywords:** Children, Auditory Processing, Test-Retest Reliability, Stimulus, Dichotic Listening Tests

## Abstract

**Purpose:**

The main objective was to develop and validate a MATLAB-based Competing Sentence Test (CST-K) and obtain preliminary normative data for this test within a small cohort of children aged 8 to 12.

**Methods:**

This study comprised two phases. Phase 1 involved developing and validating the Competing Sentence Test in Kannada (CST-K). Sentences were selected from Kannada academic textbooks of III and IV standards, recorded by a male native Kannada speaker, and evaluated by three experienced audiologists. Phase 2, which includes normative pilot estimation, was conducted on 60 right-handed children aged 8-12 without hearing difficulties. The CST-K was administered in a quiet room in the school, where the noise level ranged from 40.3 to 43.2 dBA using a laptop and soundcard, presenting thirty pairs of simple sentences with a signal-to-competition ratio of -15 dB. Test-retest reliability was assessed after three weeks. Participants were evenly distributed among four age groups: 8-8.11 years, 9-9.11 years, 10-10.11 years, and 11-11.11 years.

**Results:**

The results indicated a significant difference between the scores obtained for the right and left ears. There was also a statistically significant difference across the age groups. The test-retest reliability test showed excellent reliability for the right ear and good reliability for the left ear scores.

**Conclusion:**

A MATLAB-based Competing Sentence Test in Kannada Language was developed and validated in a recent study. The study found that scores were higher for the right ear and there was a significant age-related difference. This test can be used to evaluate binaural separation skills in Kannada-speaking children and can be included in the test battery. The study also provides normative data for the competing sentence test, which is reliable, making it a unique tool for clinicians to assess various clinical populations.

## INTRODUCTION

Central auditory processing disorder (CAPD) is a disorder in which information in the auditory nervous system is difficult to process^(1)^. It affects various aspects of auditory perception, such as sound localization and discrimination. CAPD can affect children and adults and can be caused by neurological problems, learning difficulties, aging, and peripheral hearing loss^(2)^.

Children diagnosed with auditory processing disorder (APD) often experience various difficulties. These challenges include impairments in discriminating between foreground and background sounds, limited auditory attention, deficiencies in memory, and delays in acquiring receptive language skills. Moreover, APD is characterized by subpar integrative skills, a diminished ability to sequence auditory information, challenges in phonics (the association of auditory symbols with visual symbols), and difficulties processing time-altered speech or speech exhibiting reduced redundancy^(3)^.

CAPD, characterized by heterogeneous auditory deficits, has been studied over time using numerous tests. One of the tests is the dichotic listening test, a sensitive behavioral test for assessing hemispheric function and interhemispheric transfer of information. It plays a role in assessing the development and maturation of the auditory nervous system in children and adolescents and identifying lesions of the central auditory nervous system^(1)^.

Dichotic listening is the auditory process that involves listening to both ears. It is used to reflect language laterality. In dichotic listening experiments, the typical behavioral finding is enhanced reporting of the tokens presented to the right ear, confirming the right ear advantage^(4)^. Dichotic listening is broken into two processes: binaural separation and integration. Binaural integration is the ability to perceive different acoustic signals presented to the left and right ears simultaneously. Binaural separation is the ability to perceive an acoustic message in one ear while ignoring a different acoustic message in the other ear. These tests have proven to be sensitive for children with CAPD and other developmental disorders, such as dyslexia^(5)^.

The competing sentence test (CST) determines binaural separation ability, which is the ability to process auditory information presented to one ear while ignoring the information presented to the other simultaneously^(6)^. Research has shown that children with learning disabilities have difficulty processing auditory information, which reduces their ability to cope with competing acoustic environments. The competing sentence test (Auditec) version recorded in General American English and normed based on the U.S. population has been available for clinical use—normative data for ages 5, 6, 7, 8, 9, and 10. At age 10, average scores are 100% in both ears, so it can be assumed that regular patients over age ten would achieve similar results^(7)^. In this scenario, no standardized tests that are normative in any Indian language are available. To the best of our knowledge, the competing sentence test is not available in any Indian language, including Kannada. Kannada is a language spoken in the southern state of Karnataka characterized by its distinct phonetics, intricate grammar, and broad vowel system. Unlike English, Kannada follows the Subject-Object-Verb (SOV) order and is classified as an agglutinative language, where affixes and suffixes are incorporated into root words to convey meaning and restructure sentences. Developing a CST in South Asian languages such as Kannada would be highly important for assessing children with listening difficulty at the earliest. Hence, the current study aimed to develop and validate a MATLAB-based competing sentence test in Kannada. Additionally, we aimed to determine pilot normative performance in a small cohort of children aged 8 to 12 years.

## METHOD

The current study is an observational study conducted in two distinct phases. Phase I included the development and validation of stimuli, and Phase 2 included the administration of the test to schoolchildren and test-retest reliability assessment. The study was carried out with the approval of the Institutional Ethics Committee of Kasturba Medical College and Kasturba Hospital, Manipal (IEC:358/2022), Clinical Trial Registry (CTRI/2023/01/048874), and permission was obtained from the Deputy Director of Public Instruction for data collection from the schools.

### Phase I: development and validation of stimulus

#### Stimulus selection, familiarity, and recording

A total of 250 meaningful sentences with 4 or 6 words were selected from the Kannada language academic textbooks of III and IV standards. The sentence structure of each sentence in the given text was kept in Subject-Object-Verb (SOV) format. In addition, each sentence contained a minimum of one content word that served as the target word. To ensure consistency, a word length range of 4-6 was used throughout the text. Care was taken to maintain the word length range from 4-6. Each sentence contained at least one content word used as a target word. Each sentence strictly contained the SOV format. A familiarity check was conducted at a local school in the surroundings. The school teachers were instrumental in the recruitment of five children from the 3rd and 4th grades who demonstrated exemplary academic performance. The teacher read 250 sentences to the children and asked them to rate their familiarity using a 5-point rating scale. The scale ranged from 1 to 5, with 1 representing the unknown and 5 representing the most familiar. After the familiarity check, 196 sentences had an average rating of 4 or 5 and were selected for recording. The remaining 54 sentences were ignored.

The selected sentences were recorded by a male native Kannada speaker seated comfortably and instructed to utter the sentence naturally with a neutral accent and intonation while maintaining constant vocal effort as much as possible. The stimuli were recorded using an omnidirectional head-worn microphone (PYLE PRO PMHM2) kept 6 cm from the mouth. The stimuli were subsequently recorded using Adobe Audition version 13, and the intensity of all stimuli was normalized (±3 dBSPL) to control for Root Mean Square variations in levels. The background noise in the stimuli was pruned using adaptive noise reduction in Adobe Audition. The required portions were spliced, and the edited stimuli were saved in the ‘*.wav’* format. The duration of all 196 sentences was noted, and the median duration was 2.76 seconds. Hence, stimuli between 2.5 sec and 3 sec were chosen for creating CST. After that, 120 meaningful sentences of 4 to 6 words were shortlisted from the familiar 196 sentences and selected for the final list. One or two keywords (content word) were identified for each sentence by a native speaker of Kannada. These identified keywords are considered essential for comprehending the sentences. Care was taken to avoid keywords from the final 500 msec of the sentence stimulus to overcome the unmasking effect because of unequal sentence lengths.

#### Content validation

The recorded stimuli were validated with the help of three audiologists with a minimum of 5 years of experience who were asked to evaluate the recorded stimuli. The audiologists had to rate the stimuli according to different parameters, such as intensity, appropriateness, distortion, and difficulty in identifying the tokens. The participants were asked to rate these parameters on a 5-point scale, with five being strongly recommended. Open-ended suggestions were also considered for the inclusion and exclusion of the sentence. All the experts rated all the dichotic stimuli as good quality for all the parameters (ratings between 4 and 5).

#### Development of the MATLAB program for the competing sentence and calibration setup

**Figure 1:**
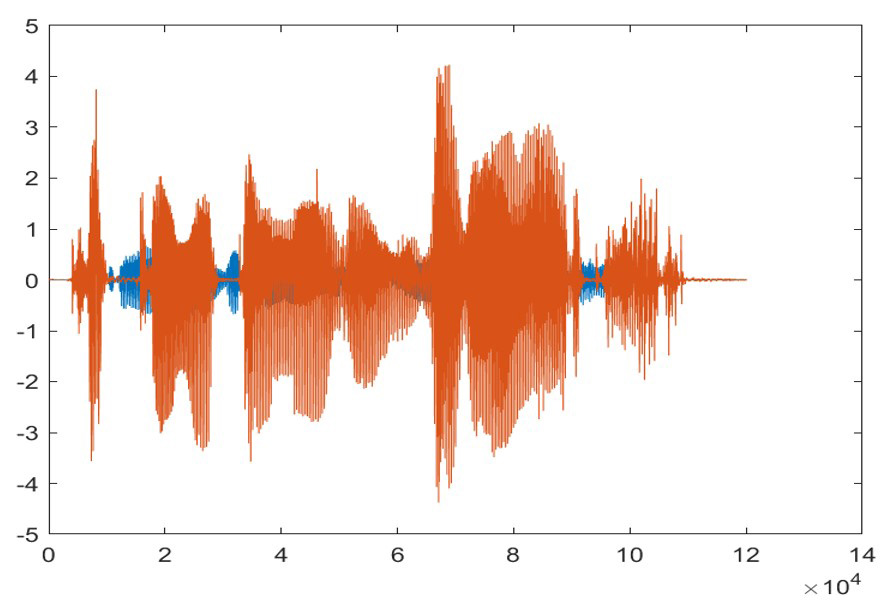
Waveform of a dichotic right competing and left target sentence stimulus

ORANGE: Right competing signal; BLUE: Left Target Signal

**Figure 2:**
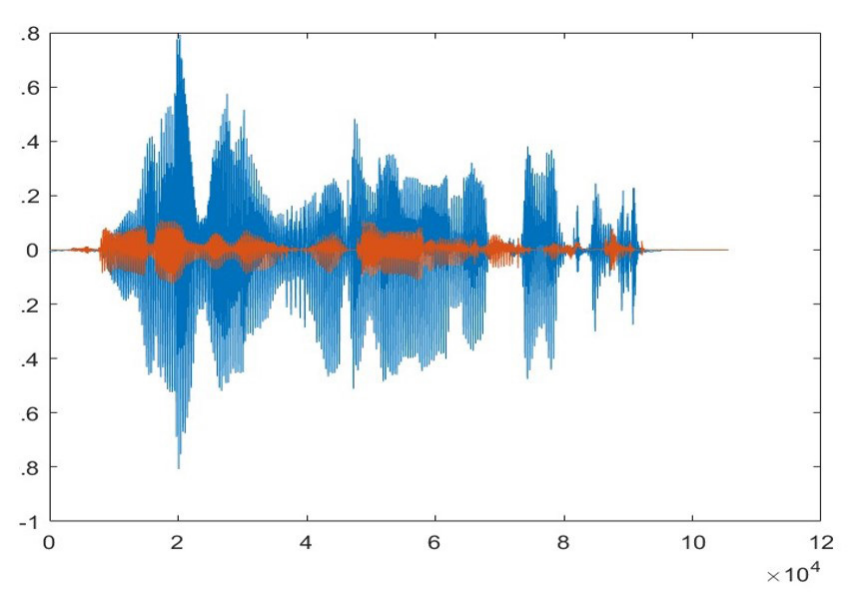
Waveform of a dichotic right target and left competing sentence stimulus

ORANGE: Right Target signal; BLUE: Left Competing Signal

The sti muli used in the study underwent RMS normalization using MATLAB to ensure comparable levels. The laptop’s sound card was adjusted to 75% amplitude to prevent distortion. The stimuli were played through Sony Wh-Ch510 Bluetooth Wireless Stereo headphones with a frequency response from 10 to 20000 Hz positioned over the 6 cc coupler at conversational levels in a quiet environment. A microphone from a Bruel & Kjaer-Type-2250 sound level meter and a Bruel & Kjaer type 4125 artificial ear were used for stimulus calibration. The overall intensity was adjusted using MATLAB code to range between 55- and 60-dB SPL. SNR-adjusted dichotic stimuli were created using MATLAB 2022b. A 'mix' function was used to store nontarget and target stimuli in the respective channels to behave like stereo stimuli. Figure 1 shows the right as competing stimuli and the left as target stimuli, while Figure 2 shows the inverse.

### Phase II: administration of tests to school children

#### Participants

All the children were in mainstream education in public schools, ranging from grades 3 to 7, with Kannada serving as their native or fluent spoken language. The participants were children from government schools from low or middle-class socioeconomic backgrounds, while those from higher socioeconomic statuses were excluded. However, a formal tool was not utilized to assess socioeconomic status, which constitutes a limitation of this study. All parents or guardians of children included in the research were informed of the study's objectives and procedures. They were invited to sign the Informed Consent Form to participate in the study. Informed consent was not waived or dismissed in this study, as we followed the standard informed consent procedures. This study included fifteen right-handed children in each age group aged 8 to 12 years. Initially, one hundred participants were screened for the study. An interview was also conducted to collect demographic details, including age, sex, and grade level. Subsequently, otoscopy was used to visualize the middle ear/ear canal. All the participants were screened for hearing loss using the Grason Stadler GSI-18 screening audiometer with RadioEar DD45 audiometric headphones and standard audiometry techniques. Testing started at 1 kHz, followed by 0.5, 4, and 2 kHz. Children were instructed to raise their hands to indicate when they heard a tone, and thresholds were recorded on a proforma sheet.

The Developmental Screening Test, developed by J Bharat Raj in 1977, was used to evaluate children's motor development, speech and language, and personal-social development. Children with ear-related complaints or a history of sensory, neurological, or psychological illness were not recruited. After screening 100 children, 60 were selected based on the exclusion criteria. These participants were evenly distributed among the four groups. The absence of notable discrepancies in scores between male and female participants justifies the consolidation of their data for analytical purposes (Table 1).

**Table 1:** Participant characteristics

**AGE GROUPS**					
	**8-8.11**	**9-9.11**	**10-10.11**	**11-11.11**	**TOTAL**
**n**	**15**	**15**	**15**	**15**	**60**
**Female/Male**	10/5	5/10	9/6	5/10	29/31
**Mean age (Standard Deviation), years**	8.37(0.358)	9.33(0.330)	10.2(0.288)	11.7(0.428)	

### Test procedure

#### Competing sentence test

**Figure 3:**
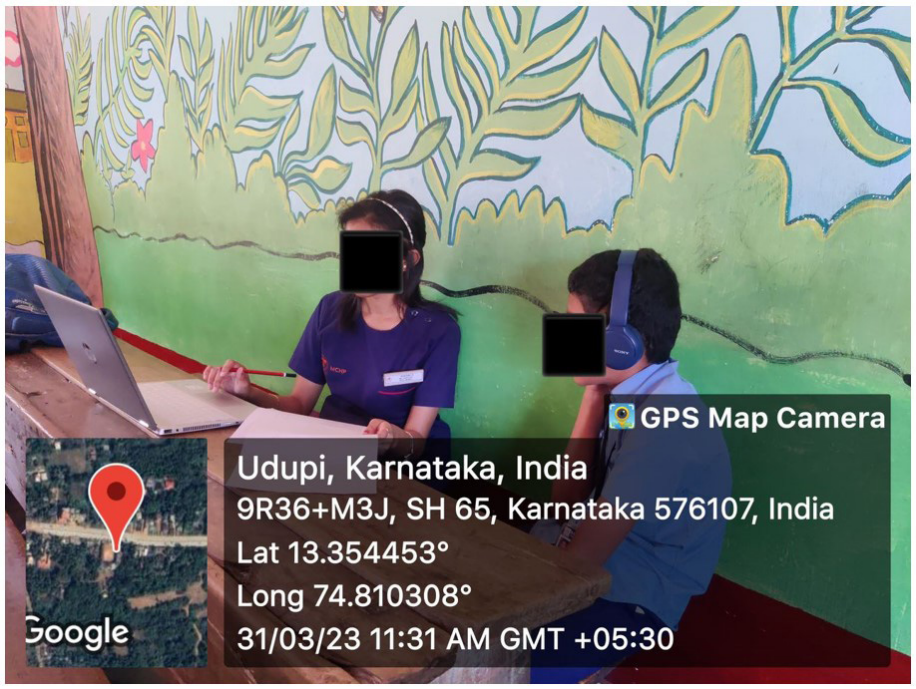
Image showing the setup of the experiment

The entire testing process occurred during school hours within the chosen educational institution. The school library or a silent classroom was selected as the testing location, where the noise levels ranged from 40.3 to 43.2 dB A. Testing was paused during interferences such as class intervals, leisure activities, and lunch intervals. The photograph is shown in Figure 3 to depict the arrangement of the setting. Two tests were conducted: hearing sensitivity and the Competing Sentence Test.

The Competing Sentence Test – Kannada (CST-K) was performed using a laptop and sound card. The laptop was paired with Sony Wh-Ch510 Bluetooth Wireless Stereo headphones with a frequency response range of 10 to 20000 Hz to present the stimulus. Thirty pairs of simple sentences, each at least six words long, were presented to both ears using a signal-to-competition ratio of -15 dB SPL.

The primary message (target sentence) was presented at 35 dB SPL in one ear (e.g., the right ear), while the competing message (competing sentence) was set at 50 dB SPL in the other ear (e.g., the left ear). Participants were instructed to focus on and repeat the target sentence while ignoring the competing sentence. The competing noise, represented by the competing sentence, was designed to interfere with the participants' comprehension of the target sentence.

Participants were required to repeat all the words in the target sentence and make an educated guess if uncertain. This procedure was repeated for each trial, alternating the ear through which the target and competing sentences were presented. Each participant took approximately 20 minutes to complete the test.

#### Scoring

The target word in that sentence was underlined on the score form. Each correctly repeated target word from the sentence was scored as 1. If the sentence was incorrect or when the message could not be repeated, a score of 0 was assigned. Ear-specific scores were obtained.

### Test-retest reliability

After a three-week interval, a sample consisting of 15 respondents was chosen from the entire group of participants based on the random draw method to assess the test-retest reliability of the CST. Identical stimuli and methodologies were employed to retest the children. The retest outcomes were compared with their previous test results using intraclass correlation (ICC) in SPSS.

### Statistical analysis

The statistical analysis was conducted using SPSS for Windows, version 16.0 from SPSS, Inc. Descriptive statistical analysis was performed, including the mean, standard deviation, and range scores. Repeated measures ANOVA was then used to determine if the observed mean differences in both ears and age groups were statistically significant. SPSS version 27 was used to calculate the intraclass correlation coefficient (ICC) to determine test-retest reliability.

## RESULTS

### Pilot normative estimation

The total correct scores for each ear were calculated separately for all the participants. Cut off scores (rounded) based on *+/- 3 Standard Deviation added in*Table 2. Ear-specific scores were obtained and analyzed across age ranges. Tables 2 and 3 show the mean and standard deviation, 95% confidence interval, and percentiles obtained for the two ears separately across age ranges.

**Table 2:** Percentile and cut off scores across age groups

**AGE**	**EAR**	**Percentiles**						
		**5**	**10**	**25**	**50**	**75**	**90**	**-3 Standard Deviation (SD)**
	**RE**	23	23.6	24	26	27	27	21
**8.0-8.11**	**LE**	23	23	24	26	26	28	21
	**RE**	24	24.6	25	26	27	27.4	21
**9.0-9.11**	**LE**	26	26	27	27	28	29	20
	**RE**	23	23	24	26	27	27	22
**10-10.11**	**LE**	23	23	23	25	26	27.4	21
	**RE**	23	23.6	24	25	27	27	24
**11.0-11.11**	**LE**	25	25	26	27	28	29	23

Caption: RE = right ear; LE = left ear; SD = Standard Deviation

**Table 3:** Mean and standard deviation for the right and left ears across all age groups

**AGE**	**EAR**	**MEAN**	**STD.DEVIATION**	**95% Confidence Interval**	
				**Lower Bound**	**Upper Bound**
8.0-8.11	RE	25.467	1.302	24.827	26.106
	LE	25.333	1.447	24.597	26.070
9.0-9.11	RE	25.467	1.552	24.827	26.106
	LE	24.933	1.624	24.197	25.670
10-10.11	RE	26.200	1.082	25.561	26.839
	LE	25.333	1.291	24.597	26.070
11.0-11.11	RE	27.467	0.915	26.827	28.106
	LE	27.000	1.309	26.263	27.737

Caption: RE = right ear; LE = left ear

The test of normality showed that most variables were normally distributed (5/8), 2 (ears) x 4 (age groups). Repeated measures ANOVA was used to test the effect of the dependent variables on the competing sentence scores. The results showed a significant main effect of the ear on the competing sentence score [F (1, 56) =10.12, p = 0.002]; Eta =0.153]. The right ear had a slight advantage in terms of competing scores over the left ear. There was also a significant main effect of age [F (3,56) =0.91, p = 0.001; Eta = 0.325]. There was no significant interaction effect [F (3, 56) = 0.915, p = 0.439; Eta = 0.047].

**Figure 4:**
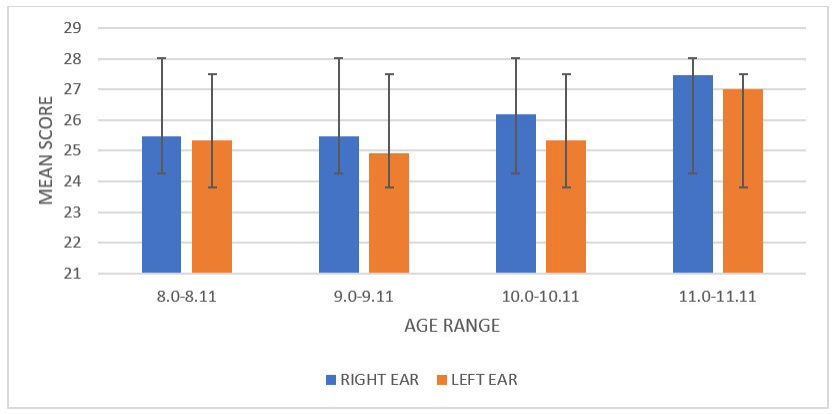
Bar graph showing the mean scores for the right and left ears across age groups

Post hoc analysis revealed significant differences between groups 8.0-8.11 and 11.0-11.11 (p=0.001) and between groups 9.0-9.11 and 11.0-11.11 (p=0.001); however, there was no significant difference between groups 8.0-8.11 and 9.0-9.11 (p=1.00). Overall, the 8- to 10-year age groups differed from the 11.0-11.11-year age group. Figure 4 shows the mean scores of both ears for all age groups.

### Test-retest reliability

The intraclass correlation coefficient (ICC) for the right ear was 0.964, with a 95% confidence interval of 0.88 to 0.98, and that for the left ear was 0.864, with a 95% confidence interval of 0.64 to 0.95 (Table 4).

**Table 4:** Test-retest reliability for the right and left ears

**EAR**	**Intra Class Correlation**	**95% Confidence Interval**	
		**Lower bound Upper bound**	
**RE**	0.964	0.887	0.988
**LE**	0.864	0.641	0.952

Caption: RE = right ear; LE = left ear

## DISCUSSION

The current study aimed to develop and validate the competing sentence test in the Kannada language. This study involved two phases. The first phase consisted of selecting Kannada sentences and testing their familiarity with children between the ages of 8 and 12. The sentences are then recorded, and their content is validated. In the second phase, a pilot study was conducted on 60 typically developing children, followed by an assessment of test-retest reliability on 15 children from the same group after two weeks.

### Stimulus selection for the Competing Sentence Test in Kannada

In this study, a pool of 250 sentences from third-grade textbooks was initially selected, and after considering familiarity and duration, 120 final sentence lists were chosen. Previous research has suggested that the difference in length between dichotic stimuli can impact final scores^(8)^. To avoid identification based purely on sentence duration, the synthetic sentence identification (SSI) test uses sentences as stimuli, with each sentence approximately the same length^(9)^. Sentence stimuli with equal word length were selected for this study, making this approach more challenging for children and providing a more sensitive measure of selective attention and cognitive abilities. This approach also reduces the likelihood of the habituation effect.

### Content validation

The stimuli were initially validated by the researchers and subsequently validated by experts. The expert content validation results also confirmed the appropriateness of the stimulus materials.

The target sentence's SNR was set at -15 dB to challenge auditory perception, as is common practice in the literature^(10)^. This ensured that the target was difficult to perceive and lacked adaptation.

During the second phase of the study, preliminary normative data were established. The descriptive statistics and normative data are shown in Tables 2 and 3. The study revealed a slight advantage in terms of right ear scores compared to left ear scores on the dichotic sentence test. However, this difference was found to be negligible and not clinically significant.

This result is expected in dichotic studies, as ceiling effects are commonly observed in directed attention tests^(11)^. Recent studies in other languages and age ranges have shown similar ceiling effects^(12)^. While the dichotic digit test is also reported to have a high ceiling effect, its clinical sensitivity has been extensively studied, and the results suggest that it is primarily useful as a cortical test and is sensitive for detecting brainstem disorders^(13)^.

In a similar direction, the dichotic sentences could be much more taxing and less predictable than the Dichotic Digit Test. Thus, clinical sensitivity may not be compromised. However, further studies are needed to validate the above findings. Furthermore, disorder-specific sensitivity and specificity scores could further improve the clinical utility of the TSVR and validate the current findings.

The present study's findings demonstrate the typical range of scores obtained by schoolchildren aged between 8 and 12 years when using dichotic sentences. The literature on dichotic test results shows that age is an essential factor influencing dichotic perception. Dichotic scores increase as age progresses, reaching a ceiling effect at approximately 12 years^(14)^. A significant reason for this difference is the maturation of Heschl’s gyrus and corpus callosum^(15)^. As in the literature, the dichotic scores in the present study showed significant improvement with age. However, there was a sharp improvement in the 11- to 12-year-old group compared to the other age groups with comparable scores.

Studies have shown that children are more affected by auditory masking than adults when it comes to recognizing speech^(16)^. However, the ability to recognize speech in noisy environments develops as children grow older, and typically doesn't mature until adolescence. Interestingly, children seem to be more vulnerable to auditory masking when the competing sound is also speech, as opposed to steady-state noise^(17)^.

Dichotic Sentence Identification scores in adults showed strong cognitive influence since DSI scores are severely impaired even in patients with mild cognitive impairment (MCI)^(18)^. A recent systematic review suggested that the DSI can predict MCI with a large effect size^(19)^. A cohort study suggested that DSI scores less than 50 are associated with an increase in the odds ratio of 4.18^(20)^. Language skills such as reading and repeating ability also influence DSI scores^(21)^. These scores may be influenced by cognitive, language, and auditory processing abilities in children. In a disordered population, any or all these components could lead to decreased DSI performance.

The test-retest reliability was calculated for both the left and right ears. Intraclass coefficient estimates, and 95% confidence intervals revealed that the reliability of the scale was “excellent” for right ear scores, while the reliability of the left ear scores was “good.”

The current study uses MATLAB, a paid platform that educational institutes subscribe to for tests. However, some platforms can execute MATLAB commands without a paid OCTAVE subscription. Validation of these platforms has yet to be performed. The data was collected in a quiet school environment but cannot be equated to a sound-treated audiometric test setup. The current norm is suitable for open field testing in schools and camps, but further verification is needed before deployment in audiometric test setups. Future directions include developing a MATLAB-based competing sentence test and assessing it on disordered populations for criterion validation. Formal working memory, cognition, and socioeconomic status assessment methods will also be integrated. Despite the robustness of our pilot study, larger sample sizes are imperative for establishing reliable cut-off scores, enhancing the clinical utility and generalizability of our findings.

### Limitations

The clinical significance of these findings can be ascertained only through the evaluation of clinical populations, such as children with central auditory processing disorder (CAPD) and other developmental disorders, such as dyslexia and functional changes in the central nervous system. Furthermore, the reliability of the study could have improved with a more representative data set obtained through a larger sample size. Moreover, the need for assessments of children's cognitive, working memory and socioeconomic status is a significant limitation that may restrict the depth of understanding of the observed phenomena.

## CONCLUSION

The current study developed and validated a MATLAB-based Competing Sentence Test in Kannada Language. The pilot results showed better scores for the right ear and a significant age-related difference. The test can assess binaural separation skills in Kannada-speaking children and can be included in the test battery. The study also provides normative data for the competing sentence test with good reliability, which adds to the study's uniqueness. Hence, this test can be used as a clinical tool to assess several clinical populations.
